# Raman spectroscopy in the detection and diagnosis of lung cancer: a meta-analysis

**DOI:** 10.1007/s10103-025-04421-y

**Published:** 2025-03-28

**Authors:** Nikita Sharma, Sowndarya Rao, Hemanth Noothalapati, Nirmal Mazumder, Bobby Paul

**Affiliations:** 1 Department of Biophysics, Manipal School of Life Sciences, Manipal Academy of Higher Education, Karnataka Manipal, 576104 India; 2 Faculty of Life and Environmental Sciences, Shimane University, 1060 Nishikawatsu-Cho, Matsue, 690-8504 Japan; 3 Department of Chemical Engineering, Indian Institute of Technology Hyderabad, Sangāreddi, Telangana 502285 India

**Keywords:** Raman spectroscopy, Lung cancer, Meta-analysis, Systematic review

## Abstract

Lung cancer is the world’s biggest cause of death related to cancer, and its dismal prognosis has been greatly exacerbated by late-stage diagnosis. Even with improvements in treatment strategies, current diagnostic techniques are frequently imprecise, especially when it comes to early-stage detection. A prospective substitute is Raman spectroscopy, which provides a non-invasive, real-time, and extremely sensitive study of biological samples. The objective of this study is to assess the diagnostic efficacy of Raman spectroscopy in the identification and diagnosis of lung cancer across a range of sample types. Nine studies that focused on Raman spectroscopy as a stand-alone diagnostic tool and met strict inclusion criteria were found through a systematic review of the literature published between 2014 and 2024. Statistical methods were used to extract, pool, and show diagnostic measures. The remarkable diagnostic accuracy of Raman spectroscopy was highlighted by its pooled sensitivity and specificity which were 98.68% and 91.81%, respectively. Serum-based research showed the strongest findings, with multivariate models such as PCA-LDA supporting specificity and sensitivity values that, in several cases, reached 100%. Diagnostic accuracy was greatly improved by models such as SVM and CNN, particularly when it came to detecting minute spectral alterations associated with cancer. Raman spectroscopy shows great promise as a lung cancer diagnostic method. However, issues including spectral data standardization, sample preparation heterogeneity and the requirement for bigger, multicentre research needs to be addressed. These results will open the door for the incorporation of Raman spectroscopy into standard clinical procedures.

## Clinical trial number

Not applicable.

## Introduction

With around 1.8 million fatalities each year, lung cancer is the primary cause of cancer-related deaths globally [22]. Lung cancer patients continue to have a poor prognosis despite improvements in therapeutic approaches, mostly because of late-stage diagnosis, which significantly lowers the chance of effective treatment results [21]. Early diagnosis is essential as it significantly raises the survival rates and also increases treatment efficacy [2, 11]. Current clinical diagnostic approaches, such as imaging and histopathology, often struggle with detecting lung cancer in its early stages, leading to delays in diagnosis and treatment [5, 16]. While conventional pathology examination remains the gold standard for detecting a variety of tumours, clinical practice needs a precise, quick, and non-invasive diagnostic tool [5]. The limitations of the current approaches serve as the impetus for the creation of novel approaches that can quickly and precisely identify and diagnose cancer in its early stages.

Spectroscopic techniques are becoming increasingly useful in biomedical research [4]. Raman spectroscopy, in particular, is emerging as a possible supplement to histopathology since it can get past the existing drawbacks. Recent developments in the field have elevated it to an extent where in vivo trials are beginning to emerge [8, 18]. The method may be able to offer automated and repeatable pathology categorization within clinically meaningful time periods, when combined with strong multivariate algorithms [8].

**Fig. 1:**
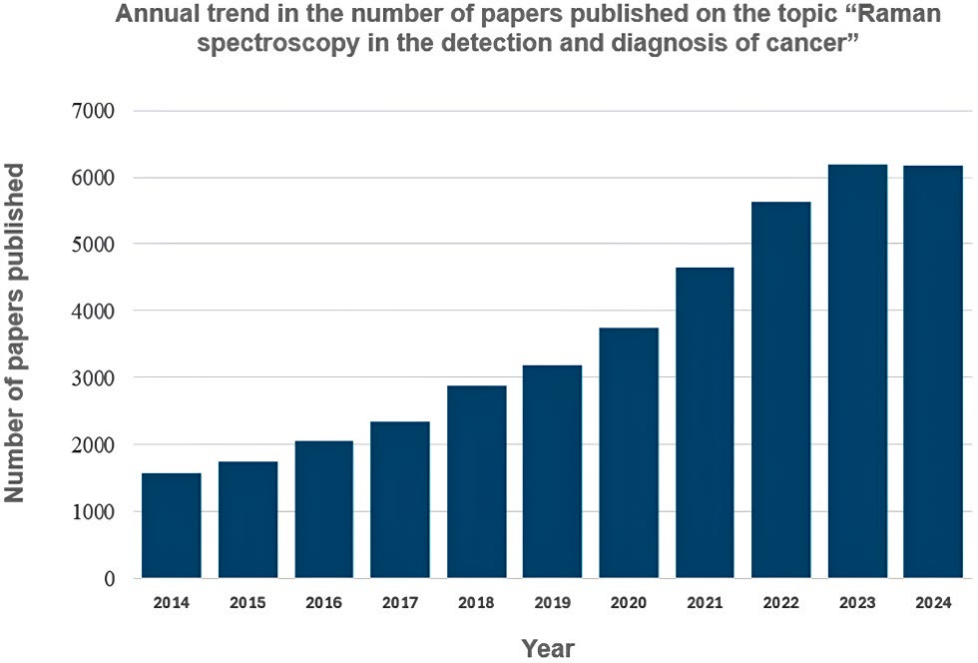
Annual trend in the number of papers published on the topic “Raman spectroscopy in the detection and diagnosis of cancer”

Since each biological sample has its own distinctive chemical makeup, a sample specific spectral fingerprint, or “Raman spectrum”, can be generated by using Raman spectroscopy, which is a type of spectroscopic method that can investigate the vibrational patterns linked to chemical bonds in a given sample. Multiple bands at varied frequencies that are indicative of a given molecule’s structural characteristics and functional groups can be found in this spectrum. Each biological sample has a unique chemical composition, leading to distinct vibrational patterns when analysed using Raman spectroscopy. These spectral patterns provide detailed molecular information about proteins, carbohydrates, lipids, and nucleic acids present in tissues and cells. By detecting these biochemical variations, Raman spectroscopy can effectively differentiate cancerous and non-cancerous cells and tissues [4]. Proteins, lipids, carbohydrates, and nucleic acids have the highest molecular vibrational patterns in the “fingerprint” region, which contains the majority of peaks for biological samples [4]. The use of Raman spectroscopy in the detection of cancer has been the subject of multiple studies throughout the last ten years, with promising findings (Fig. 1). For the detection and diagnosis of cancer, several forms of Raman spectroscopy are more frequently employed. Surface-Enhanced Raman Spectroscopy (SERS) significantly enhances Raman signals by using nanostructured metallic surfaces, making it highly sensitive for detecting low-concentration biomarkers. However, reproducibility challenges due to variability in nanoparticle synthesis remain a major limitation [3]. Tip-Enhanced Raman Spectroscopy (TERS) integrates scanning probe microscopy with Raman spectroscopy, allowing nanoscale spatial resolution, making it suitable for subcellular imaging. While this provides high sensitivity, instrumentation complexity and long scanning time limits its feasibility in clinical settings [6]. Coherent Anti-Stokes Raman Scattering (CARS) is a nonlinear Raman technique that enables label-free, high-speed imaging of biological tissues with high molecular specificity. Despite its rapid acquisition capability, CARS suffers from background noise issues, which can lead to spectral distortions [7, 24]. Because of its excellent specificity and sensitivity, SERS is one of the most commonly employed methods in the detection of early-stage cancer [20].

Because of its adaptability, Raman spectroscopy can be used with a number of biological samples, such as serum, saliva, and tissues [23]. The diagnosing abilities of Raman spectroscopy have been further improved by developments such as the incorporation of machine learning algorithms [20]. In this meta-analysis, machine learning and statistical algorithms were key to analysing Raman spectral data. Principal Component Analysis-Linear Discriminant Analysis (PCA-LDA) reduces data dimensionality and enhances class separation, ideal for distinguishing cancerous from non-cancerous samples [27]. Orthogonal Partial Least Squares-Discriminant Analysis (OPLS-DA) improves interpretability by isolating predictive variations, offering high accuracy in complex datasets [13]. Support Vector Machine (SVM) identifies optimal hyperplanes for classifying high-dimensional spectral data [20], while Convolutional Neural Network (CNN) automatically extracts hierarchical features, detecting subtle biochemical changes for improved diagnostic precision [14]. While PCA-LDA and OPLS-DA are ideal for smaller datasets due to their simplicity and interpretability, SVM and CNN offer superior performance in larger, more complex datasets due to their ability to model non-linear relationships and intricate spectral patterns. Despite these encouraging advancements, there are still a number of obstacles to cross before the clinical application of Raman spectroscopy comes into major practice for the diagnosis of lung cancer. Major challenges include the lack of standardized protocols in Raman spectral acquisition, preprocessing, and analysis. Variations in factors such as laser power, source wavelength, sample thickness, and normalization techniques can introduce inconsistencies in spectral outputs, making cross-study comparisons difficult. The development of uniform analytical frameworks is necessary to ensure reproducibility and clinical translation of Raman spectroscopy for lung cancer diagnostics [23].

This study aims to evaluate the diagnostic performance, potential applications, and current limitations of Raman spectroscopy in lung cancer detection by combining results from research published between 2014 and 2024. We hope that this analysis will shed light on future research initiatives and make it easier to incorporate Raman spectroscopy into clinical practice for the diagnosis of lung cancer.

## Method and methodology

### Data collection and search strategy

Using the terms “Raman spectroscopy”, “detection/diagnosis”, and “lung cancer”, a thorough search was executed across four electronic databases– PubMed, Web of Science, Scopus, and Embase in September 2024. To maximize retrieval, the “all fields” filter was used in the search. All pertinent studies that were found by the search were exported in CSV format together with metadata such as the title, authors, journal, DOI, and year of publication. To facilitate processing, these datasets were combined into a single Excel file. The review process was organized using the PRISMA procedure, which complies with accepted standards for meta-analyses [17]. Since this meta-analysis is based entirely on previously published studies that already obtained appropriate ethical approvals, no new data involving human participants were collected or analysed. Thus, ethical approval was not necessary. The literature search yielded a total of 551 articles. Records identified individually from these different databases are PubMed = 114, Embase = 135, WoS = 139 and Scopus = 163.

### Deduplication

Microsoft Excel was used to find and eliminate duplicate entries that came from overlapping database records. The dataset in the combined excel file was sorted alphabetically, and Conditional Formatting → Highlight Cell Rules → Duplicate Values was applied to automatically flag overlapping records based on title, and author names. These flagged duplicates were then manually verified and removed to ensure accuracy. This process ensured that each study was included only once in the subsequent analysis. Out of 551 articles, 303 unique articles were shortlisted after removing redundancy using MS Excel.

### Study selection process

Following deduplication, studies underwent rounds of sequential screening to guarantee eligibility. Initial filtering was done based on the relevance of the study titles and the abstract. Finally, a thorough full-text assessment was done to determine which papers were suitable.

### Inclusion and exclusion criteria

A systematic selection process was applied to identify high-quality studies for meta-analysis. The screening process included title and abstract screening, full-text assessment, and strict adherence to predefined inclusion and exclusion criteria.

Studies were included if they were primary research articles published in peer-reviewed journals between 2014 and 2024, focusing on the application of Raman spectroscopy as a primary tool for lung cancer detection, with spectroscopic variations such as SERS, TERS, and CARS also considered. Although these techniques differ in their signal enhancement mechanisms, resolution, and application methodologies, they are all variants of Raman spectroscopy that rely on the same fundamental principle of inelastic light scattering to analyse molecular vibrations.

Only studies that applied machine learning-based spectral classification or statistical models to analyze Raman spectral data were considered. In addition, studies were required to report key diagnostic performance metrics, including sensitivity and specificity values. Only studies that demonstrated sensitivity and specificity of 80% or higher were retained, as this threshold ensured that the reported methodologies provided clinically relevant accuracy. Furthermore, only studies that provided clear experimental methodologies, detailing Raman instrumentation, spectral preprocessing techniques, and classification models, were considered suitable for inclusion. Studies were excluded if they were review articles, meta-analyses, case reports, or conference abstracts. Publications in languages other than English were not considered. In terms of diagnostic approach and study design, studies that combined Raman spectroscopy with other imaging or histopathological techniques, were excluded to maintain focus on Raman spectroscopy as a standalone method. To maintain statistical rigor, studies with insufficient sample sizes, defined as fewer than 40 cases, were not included in the final selection. Finally, the quality and reproducibility of the selected studies were critically assessed.

**Fig. 2:**
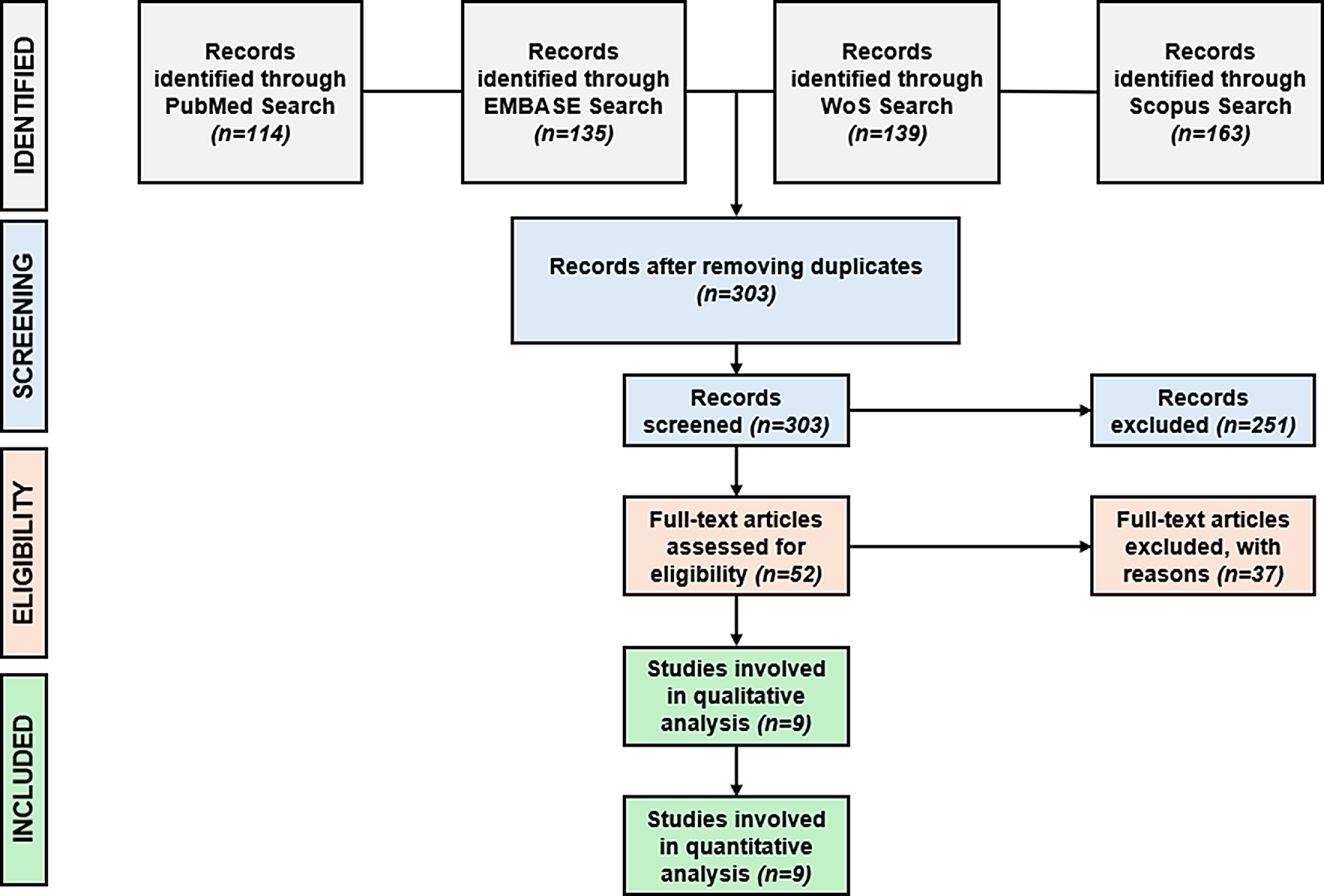
Progression of information across the different stages of a systematic review (Prisma chart)

After reviewing the 303 articles based on their title and abstract, 52 records were included further in the studies and were assessed for their eligibility and 9 articles were finally selected and included in the meta-analysis considering the inclusion and exclusion criteria. Studies that lacked full-text availability or did not provide sufficient raw spectral data for independent validation were excluded. Additionally, studies with a high risk of bias, such as those missing statistical validation or lacking independent test sets for machine learning models, were removed to ensure that only high-quality, reproducible studies were included in the final meta-analysis. It is important to note that some papers did not provide necessary diagnostic performance metrics such as sensitivity and specificity, and other necessary criteria for eligibility in the abstract or initial sections and therefore, it was only possible to evaluate and exclude these studies during the full-text assessment. Figure 2 presents the PRISMA flowchart detailing the systematic review process, from initial identification to final selection.

### Statistical analysis and visualization

We carried out a thorough statistical study to evaluate the diagnostic efficacy of Raman spectroscopy in identifying lung cancer. Pooled estimates were derived, and consistency and potential bias were assessed using the sensitivity and specificity values reported in the included studies. Each study’s sensitivity, specificity, and sample size were extracted. To guarantee consistent comparability between trials, the extracted data were normalized using min-max normalization before inclusion in the meta-analysis. Sensitivity refers to the ability of Raman spectroscopy to correctly identify lung cancer cases (true positives), while specificity indicates its ability to correctly classify non-cancerous cases (true negatives). Higher sensitivity ensures minimal false negatives, which is critical for early detection, whereas higher specificity reduces false positives, improving diagnostic reliability. Additionally, confidence intervals (CIs) were calculated using reported data in cases where they were not given to express the range within which the true sensitivity and specificity values are expected to lie with 95% confidence. A narrower CI indicates more precise estimates, whereas a wider CI suggests greater variability in the study results. To graphically compare the performance indicators across experiments, a heatmap was created. In order to find trends and group studies with comparable diagnostic accuracy, hierarchical clustering was used. To unify the scale across measures and research, the heatmap was created using normalized values. To assess possible publication bias for sensitivity and specificity, funnel plots were created. To account for study heterogeneity, pooled estimates of sensitivity and specificity were computed using a random-effects model. The random-effects model was chosen over the fixed-effects model because the included studies exhibit inherent variations in methodology, sample types, Raman spectroscopic techniques, and statistical models used for analysis. Unlike the fixed-effects model, which assumes that all studies estimate a common true effect size, the random-effects model accounts for potential heterogeneity by allowing each study to have its own true effect, drawn from a distribution of effects. To show the estimates from each study, together with their 95% CIs and the combined estimates for sensitivity and specificity, a forest plot was created. All statistical analyses were performed using R software. The heatmap was generated using the pheatmap package in R, which facilitated hierarchical clustering of performance metrics. Funnel plots were constructed using the ggplot2 package, and the forest plot was generated using the meta package.

## Results and discussion

From an initial pool of 551 articles screened, 9 studies met the inclusion criteria. These selected studies provide a comprehensive overview of how Raman spectroscopy can be incorporated into cancer diagnosis. The selected studies demonstrate how Raman spectroscopy, and specifically, SERS, can be used in a variety of biological sample types, such as serum, saliva, tissue, and plasma exosomes, for the detection of lung cancer. The majority of the research was conducted using serum-based studies, which demonstrated the most reliable and robust diagnostic accuracy with specificities ranging from 83.3 to 100% and sensitivities that reached up to 100%. For example, one study that used the large-laser-spot SERS in conjunction with Principal component analysis and linear discriminant analysis (PCA-LDA) model with a total sample of 110 people comprising 50 lung cancer patients, and 60 controls, demonstrated a remarkable diagnostic precision by identifying between cancerous and non-cancerous serum samples with 100% sensitivity and specificity [27]. Further confirming the validity of serum as a diagnostic medium, another study conducted an OPLS-DA-based analysis on a cohort of 190 participants, consisting of 108 lung cancer patients and 82 healthy individuals. The study achieved a sensitivity of 98.1% and specificity of 97.6%, successfully identifying biochemical changes in serum biomarkers associated with lung adenocarcinoma progression [13]. Additionally, [25] developed a SERS-based approach utilizing Ag-NPs/PSi enhancement for 100 serum samples divided equally between lung cancer patients and healthy controls. This study employed Principal Component Analysis (PCA), achieving 100% sensitivity and 90% specificity, highlighting the potential of nanoparticle-assisted signal enhancement for improving serum-based diagnostics. Lastly, further reinforcing the efficacy of serum analysis, [10] conducted an investigation on 69 serum samples, consisting of 51 lung cancer patients and 18 controls, incorporating Partial Least Squares Discriminant Analysis (PLS-DA). This study achieved 100% sensitivity and 83.33% specificity.

Saliva-based diagnostics, though patient-friendly and non-invasive, showed inconsistent results across different studies. Based on the statistical or machine learning models used, the sensitivity ranged from 91.2 to 100%, while the specificity had its range from 80.2 to 100% [12, 19]. Saliva’s potential as a reliable diagnostic medium was demonstrated by a study that analysed 127 saliva samples, with 61 lung cancer patients and 66 healthy individuals and achieved 100% sensitivity and specificity using SVM-based classification [19]. In contrast, a study conducted on 78 saliva samples, comprising 52 cancerous and 26 non-cancerous cases, and reported 91.2% sensitivity and 80.2% specificity [12]. Glycoproteins and DNA peaks were two important spectral indicators found in saliva that were reliably connected to cancer. Although, saliva-based techniques have potential for screening large populations, some studies have found that their intermediate specificity indicates need for additional methodological improvement to reduce false positives.

Tissue-based investigations, while highly accurate, are less scalable due to their invasive nature. A controlled study examined 46 lung tissue samples, evenly distributed between cancerous and non-cancerous cases, and applied tissue-slice SERS with PCA-LDA for spectral differentiation. The study reported a sensitivity and specificity of 95.7 [26]. Similarly, single-point Raman spectroscopy demonstrated that it was suitable for real-time intraoperative diagnoses with 94% sensitivity and 80% specificity across a more extensive sample set of 197 [9]. Tissue-based approaches excel in confirming diagnostics but are less suited for large-scale screens.

Artificial intelligence methods like Support Vector Machines (SVM) and Convolutional Neural Networks (CNN) were incorporated into plasma exosome-based research. A study analysed 40 plasma samples, equally split between lung cancer patients and healthy controls, and reported 83.3% sensitivity and specificity using CNN-SVM classification models [14]. Despite promising results, the small sample size suggests the need for larger validation studies to establish the clinical reliability of plasma-based Raman spectroscopy for lung cancer detection.

**Fig. 3:**
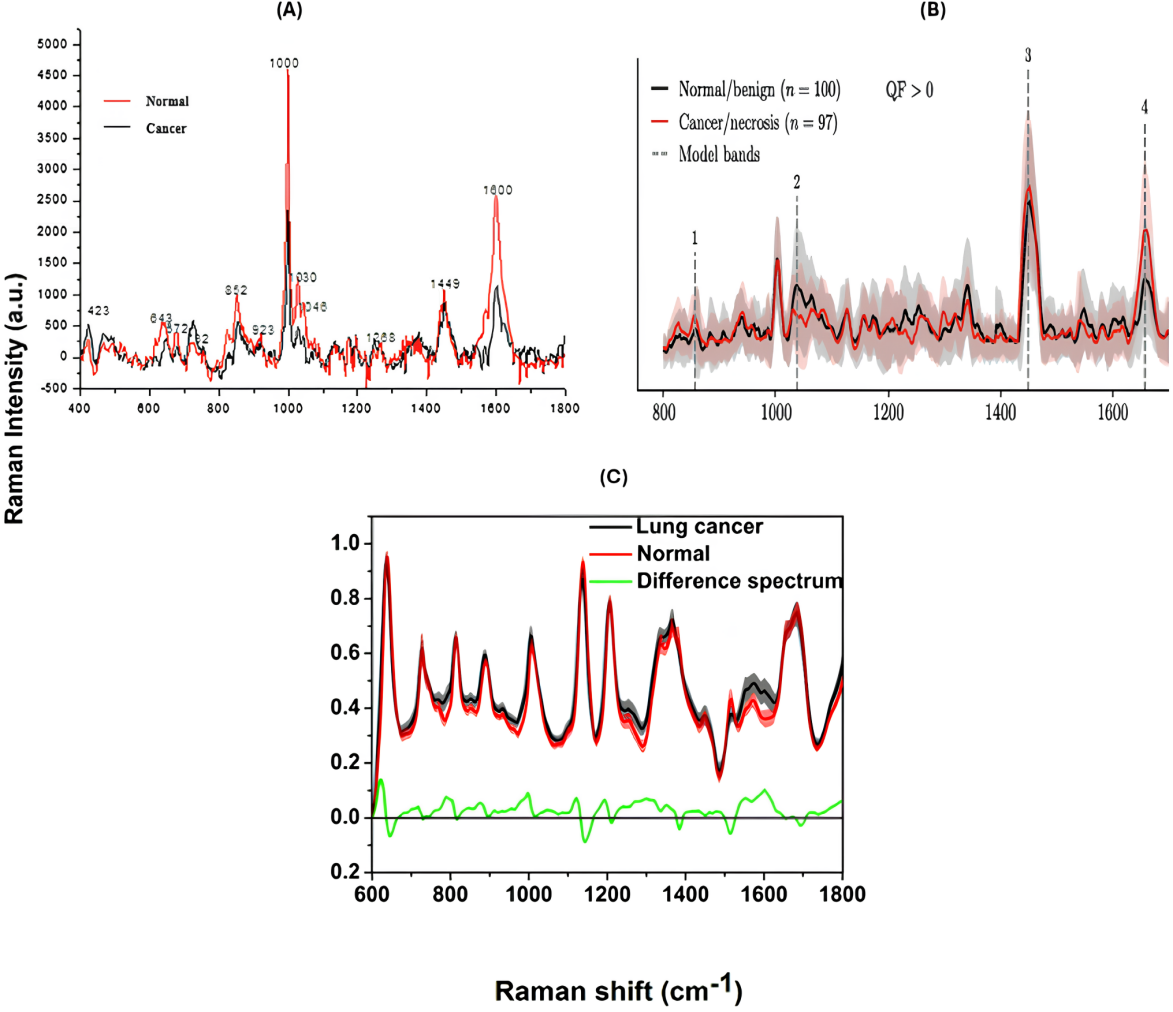
Comparative Raman spectra of lung cancer and normal tissues from three individual studies. (**A**) Raman spectra showing peaks for normal (black) and cancerous (red) tissues, with labelled wavenumbers corresponding to respective molecular vibrations. (**B**) Raman spectra demonstrating the spectral distinction between benign/normal and cancerous/necrotic tissues, with the shaded areas displaying standard deviations and dashed lines indicating key model bands. (**C**) The variations between lung cancer(black) and normal (red) tissues are highlighted, with the green line representing the calculated difference spectrum [9, 19, 25]

Some spectral biomarkers were consistently reported to be important indications of malignancy across all the investigations. Most of the samples showed strong Tryptophan (~ 1365 cm^− 1^), Protein associated peaks — amide III band (~ 1217 ^− 1^– 1273 cm^− 1^) and amide I band (~ 1600 cm^− 1^– 1683 cm^− 1^), collagen and phospholipids associated peaks(~ 1445 cm^− 1^), and phenylalanine peaks (~ 1004 cm^− 1^), highlighting their vital role in cancer detection (as shown in Fig. 3).

Overall, the results from the individual studies demonstrate that Raman spectroscopy provides consistently high sensitivity and specificity across various sample types, particularly in serum-based analyses. While saliva and plasma-based approaches show promise for non-invasive diagnostics, variability in specificity highlights the need for improved standardization. Furthermore, the integration of machine learning algorithms has played a crucial role in enhancing diagnostic accuracy.

The variations in specificity for saliva and plasma-based Raman spectroscopy are primarily due to biological and technical factors. Saliva’s biochemical composition is highly variable, influenced by hydration levels, oral health, and diet, leading to inconsistent spectral outputs [1]. Similarly, plasma-based techniques face challenges in isolating and purifying exosomal biomarkers, contributing to spectral variability [15]. Standardization through consistent sample collection protocols, improved exosome isolation, and advanced preprocessing algorithms can mitigate these issues. In contrast, serum-based techniques outperform saliva and plasma due to serum’s stable composition and standardized protocols, resulting in higher diagnostic specificity and sensitivity [13]. Tissue-based techniques, although invasive, provide direct access to tumor-specific biomarkers, offering superior diagnostic accuracy [9].

These results show how adaptable Raman spectroscopy is as a lung cancer detection technique, offering important new information about how well it works with various sample types and statistical models. The scalability of serum-based methods and the availability of saliva and plasma-based techniques underscore the possibility of incorporating Raman spectroscopy into both invasive and non-invasive clinical workflows, even though tissue-based techniques continue to be the gold standard for accuracy. To fully utilize Raman spectroscopy in clinical practice, further developments in spectral standardization, machine learning integration, and extensive validation research is required. The details of each individual study have been listed and tabulated in Table 1.

**Table 1:** Summary of studies included in the meta-analysis evaluating Raman spectroscopy for lung cancer detection and diagnosis. The table presents key parameters of each study, including the title, type of Raman spectroscopy employed, laser wavelength, laser power, sample type, sensitivity, specificity, machine learning or statistical models used and the total sample size

Sl. No.	Title	Type of RS used for detection/ diagnosis	Laser Wavelength	Power	Sample type	Sensitivity	Specificity	Machine learning/ statistical model used	Total sample size
1.	Artificial intelligence-based plasma exosome label-free SERS profiling strategy for early lung cancer detection	SERS-surface enhanced Raman spectroscopy	785 nm laser	0.7mW	Human Plasma	83.3%	83.3%	Convolutional Neural Network-Support Vector Machine	40
2.	Label-free and stable serum analysis based on Ag-NPs/PSi surface-enhanced Raman scattering for non-invasive lung cancer detection	SERS	532 nm laser	0.48mW	Human serum	100%	90%	Principal Component Analysis	100
3.	Label-free diagnosis of lung cancer with tissue-slice surface-enhanced Raman spectroscopy and statistical analysis	SERS	532 nm laser	0.5mW	Human lung tissues	95.7%	95.7%	Principal Component Analysis-Linear Discriminant Analysis	46
4.	Label-free surface-enhanced Raman spectroscopy analysis method for liquid biopsy and its application in serum-based lung cancer diagnosis and classification	Large laser spot SERS	633 nm laser	1.35mW	Serum	100%	100%	Principal Component Analysis-Linear Discriminant Analysis	110
5.	Label-free surface-enhanced Raman spectroscopy for diagnosis and analysis of serum samples with different types lung cancer	SERS	532 nm laser	4.73mW	Serum	100%	83.33%	Partial Least Squares– Discriminant Analysis	69
6.	Label-free surface-enhanced Raman spectroscopy of serum based on multivariate statistical analysis for the diagnosis and staging of lung adenocarcinoma	SERS	785 nm laser	45mW	Human serum	98.1%	97.6%	Orthogonal Partial Least Squares- Discriminant Analysis	190
7.	New method of lung cancer detection by saliva test using surface-enhanced Raman spectroscopy	SERS	785 nm laser	0-300mW	Human saliva	100%	100%	Support Vector Machine	127
8.	3D plasmonic hexaplex paper sensor for label-free human saliva sensing and machine learning-assisted early-stage lung cancer screening	SERS	785 nm laser	4 mW	Human saliva	91.2%	80.2%	Linear Regression	78
9.	Sub second lung cancer detection within a heterogeneous background of normal and benign tissue using single-point Raman spectroscopy	single-point fingerprint (800 to 1700 cm^− 1^) Raman spectroscopy	785 nm	150 mW	Human lung tissues	94%	80%	Support Vector Machine (QF > 0.4)	197

**Fig. 4:**
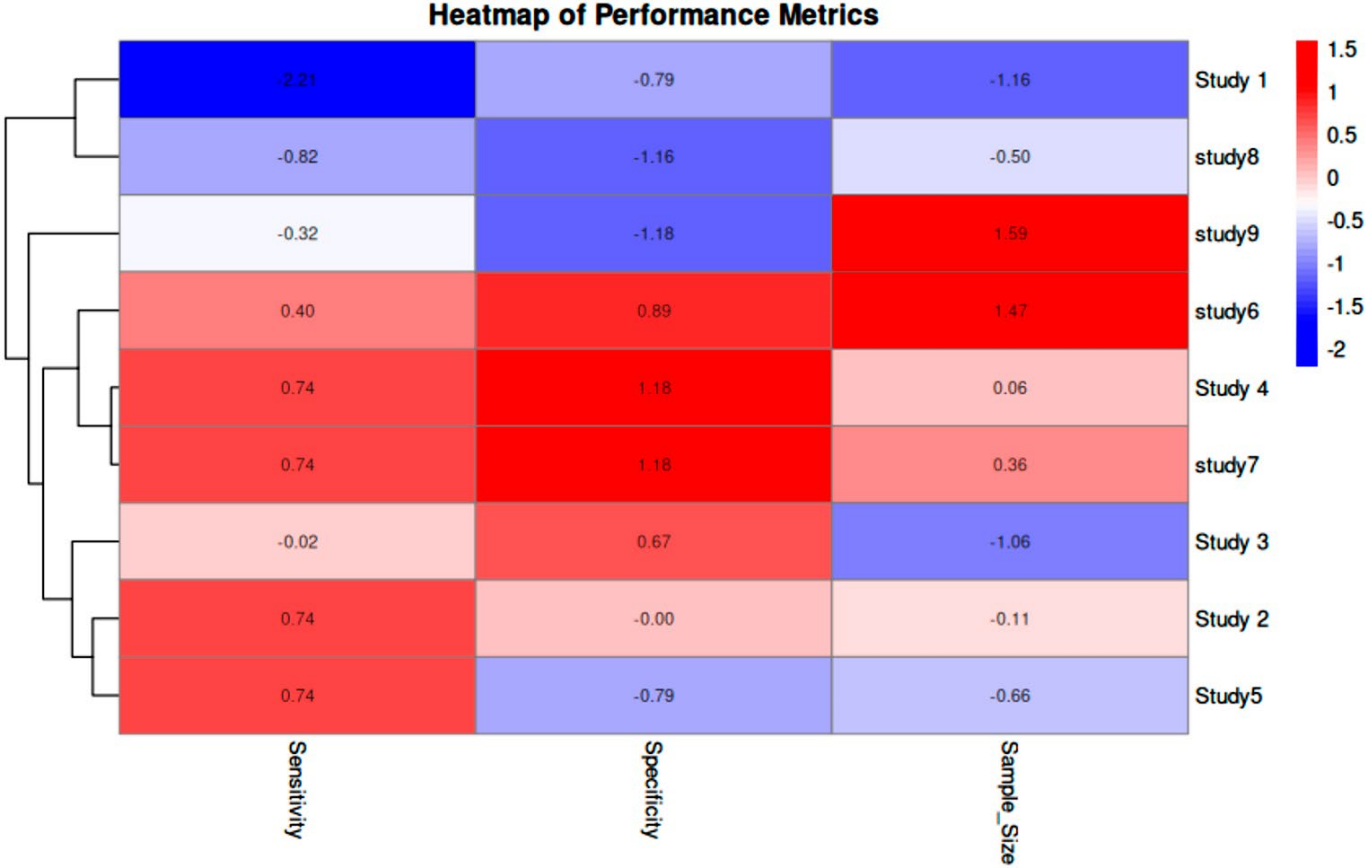
Heatmap of diagnostic performance metrics (sensitivity, specificity and sample size) across the studies included in the meta analysis. The color scale demonstrates normalized figures, with red specifying higher performance metrics and blue representing lower values. Hierarchical clustering was employed to organize the studies based on similarities in diagnostic performance (sensitivity and specificity). The clustering visually highlights how studies with higher sensitivity and specificity (e.g., studies 4 and 7) tend to group together, whereas studies with lower values (e.g., study 1) are separated, allowing for easier interpretation of performance trends across studies

**Fig. 5:**
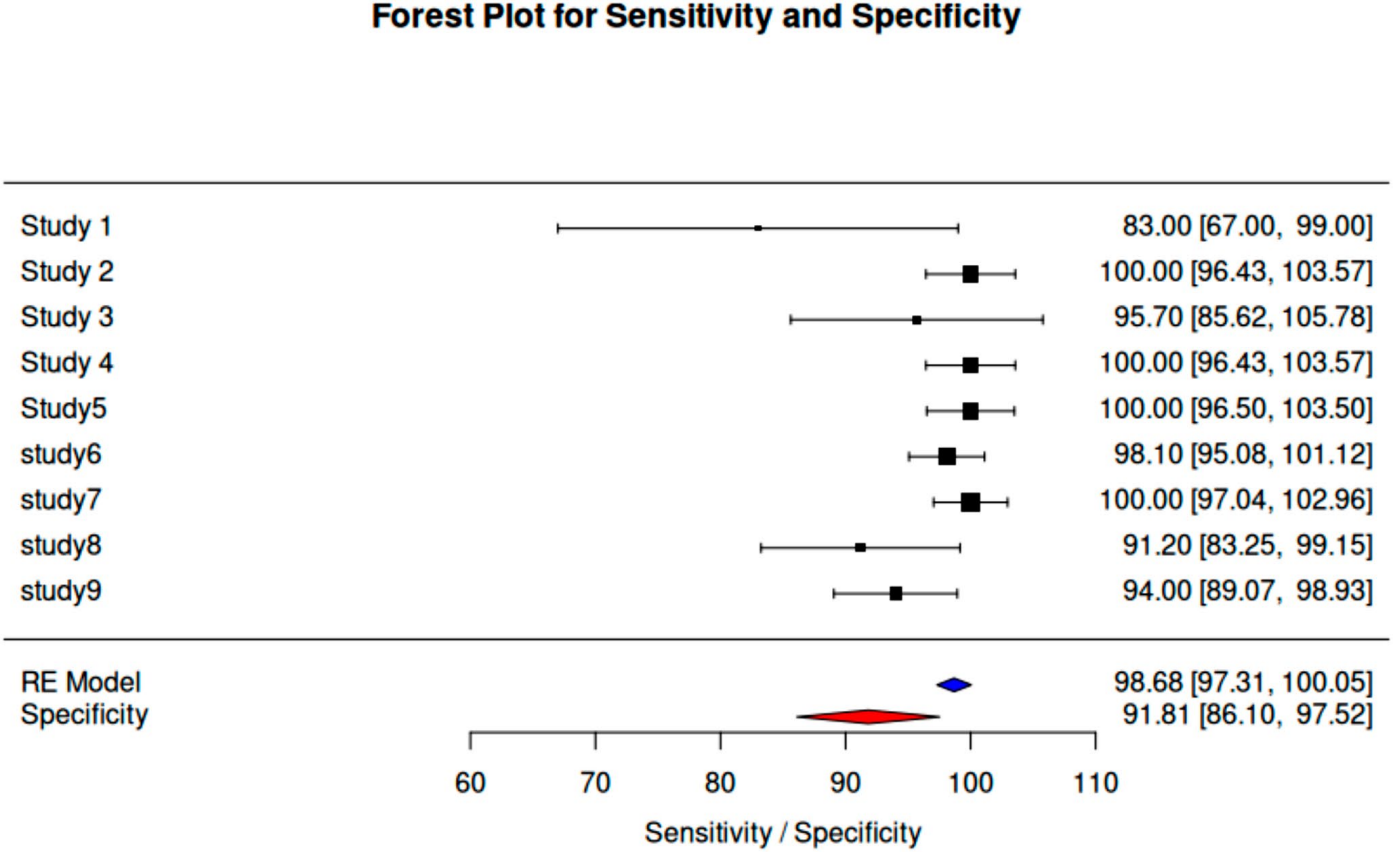
Forest plot summarizing and displaying the sensitivity and specificity in the diagnosis and detection of lung cancer across nine studies. Horizontal lines indicate the 95% confidence intervals for each study. The pooled sensitivity is 98.68% (95% CI: 97.31–100.5) and the pooled specificity is 91.81% (95% CI: 86.10-97.52), represented by the diamond markers at the bottom of the plot. This plot highlights the variability in specificity across studies, with most achieving high sensitivity values

**Fig. 6:**
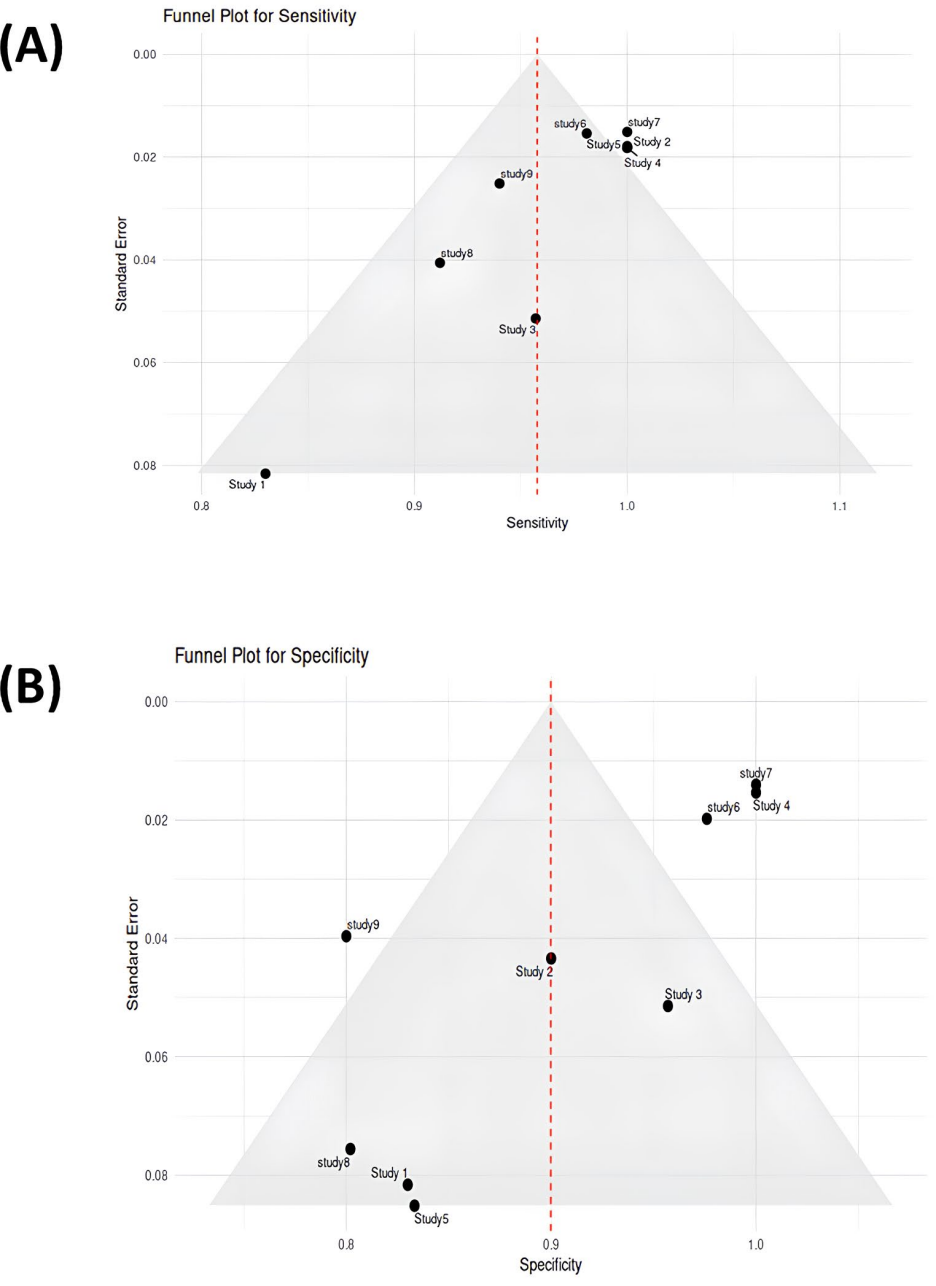
Funnel plots illustrating the sensitivity and specificity of Raman spectroscopy across nine studies of the meta-analysis. The x-axes represent sensitivity and specificity values, respectively while the y-axes demonstrate standard error. Panel (**A**) illustrates the funnel plot for sensitivity, while panel (**B**) displays the funnel plot for specificity. Each black dot represents an individual study, plotted according to its effect size (sensitivity or specificity) and standard error. The Gray triangular region indicates the expected distribution of studies in the absence of publication bias. The red dashed lines highlight the pooled sensitivity (98.68%) and pooled specificity (91.81%) values. The symmetrical distribution of studies in the sensitivity plot points towards low publication bias, with most studies clustering near the pooled estimate. In contrast, the specificity plot displays slight asymmetry, indicating mild publication bias or heterogeneity. The highlighted variability might be a result of differences in methodologies, sample types, or statistical approaches

A heatmap, funnel plots and a forest plot were used to graphically summarize the meta-analysis findings. Studies with high sensitivity, specificity, and bigger sample sizes are clustered together in the heat map, indicating that reliable study designs support reliable diagnostic results. The colour scale represents normalized values, where red indicates higher performance metrics and blue signifies lower values (Fig. 4). The forest plot visualizes the sensitivity and specificity for diagnosing and detecting lung cancer across nine studies. Horizontal lines represent the 95% confidence intervals for each study. The pooled sensitivity is 98.68% (95% CI: 97.31–100.5), and the pooled specificity is 91.81% (95% CI: 86.10–97.52), depicted by diamond markers at the bottom of the plot. This visualization highlights the variation in specificity across studies, while most studies demonstrate consistently high sensitivity (Fig. 5). Although, there is some asymmetry in the specificity plot, which shows heterogeneity in some studies, the sensitivity and specificity funnel plots show low publication bias overall. The x-axes represent sensitivity and specificity values, while the y-axes indicate standard error. Red dashed lines mark the pooled sensitivity (98.68%) and pooled specificity (91.81%) (Fig. 6(A) and (B)).

## Conclusion

This meta-analysis accentuates the promise of Raman spectroscopy as a precise diagnostic method for lung cancer, with pooled sensitivity and specificity values of 98.68% and 91.81%, respectively. The most dependable and scalable techniques were those based on serum, but non-invasive diagnostics using saliva and plasma showed promise, especially for large-scale screenings. Although, tissue-based techniques are quite precise, their invasive nature makes them more appropriate for confirmatory or intraoperative applications. By making it possible to detect minute spectral changes linked to cancer, the incorporation of machine learning models like SVM and CNN has greatly improved the diagnostic accuracy of Raman spectroscopy. However, the clinical adoption of Raman spectroscopy faces several challenges, including the lack of standardized spectral acquisition protocols, variability in sample preparation methods, and limited large-scale, multicenter validation studies. Addressing these issues will require the development of uniform protocols for spectral data collection and preprocessing, ensuring consistency across studies. Additionally, the integration of robust machine learning algorithms can help manage biological variability and enhance diagnostic accuracy. Conducting multicenter trials with diverse patient populations will be crucial for validating the reliability of Raman spectroscopy in routine clinical workflows. Overcoming these challenges will pave the way for its effective incorporation into standard diagnostic procedures. In conclusion, by enhancing early diagnosis and treatment results, Raman spectroscopy provides a revolutionary approach to lung cancer diagnostics.

## Data Availability

The data can be available upon request to corresponding author.
